# Efficacy, Feasibility, and Utility of a Mental Health Consultation Mobile Application in Early Care and Education Programs

**DOI:** 10.3390/children12060800

**Published:** 2025-06-19

**Authors:** Ruby Natale, Yue Pan, Yaray Agosto, Carolina Velasquez, Karen Granja, Emperatriz Guzmán Garcia, Jason Jent

**Affiliations:** 1 Mailman Center for Child Development, University of Miami Miller School of Medicine, Miami, FL 33136, USA; yagosto@med.miami.edu (Y.A.); cxv308@med.miami.edu (C.V.); kxg495@med.miami.edu (K.G.); eguzmangarcia@med.miami.edu (E.G.G.); jjent@med.miami.edu (J.J.); 2 Department of Public Health Sciences, University of Miami Miller School of Medicine, Miami, FL 33136, USA; panyue@med.miami.edu

**Keywords:** childcare, mental health consultation, mobile health, artificial intelligence, young children, classroom practices, mobile childcare app

## Abstract

**Background/Objectives:** Preschool children from low-income, ethnically diverse communities face disproportionate rates of behavioral challenges and early expulsion from early care and education (ECE) programs. This study evaluated the efficacy, feasibility, and utility of Jump Start on the Go (JS Go), a bilingual, AI-enabled mobile application. JS Go is designed to deliver a 14-week early childhood mental health consultation model in under-resourced ECE settings. **Methods**: This mixed-methods study compared JS Go to the standard in-person Jump Start (JS) program. Participants included 28 teachers and 114 children from six centers (three JS Go, three JS). Quantitative measures assessed teacher classroom practices and child psychosocial outcomes at baseline and post-intervention. App usability and acceptability were only evaluated post-intervention. Seven semi-structured interviews were conducted post-intervention with JS Go directors/teachers to assess the app’s feasibility for implementing the four program pillars: safety, behavior support, self-care, and communication. **Results**: JS Go was more effective than JS in promoting teacher classroom practices related to behavior support and resiliency. Both programs were similar in improving children’s protective factors and reducing internalizing behaviors, with consistent effects across English and Spanish-speaking children. Teachers rated the JS Go app with high acceptability, though predicted future usage showed greater variability. Rapid qualitative analysis showed that participants found the app easy to use, frequently accessed its resources, and considered it helpful for reinforcing key strategies across the four program pillars. **Conclusions**: JS Go is a novel approach to providing mental health consultation. It represents a promising mobile adaptation of the established JS consultation model, with important implications for future practice and research.

## 1. Introduction

Early care and education (ECE) programs play a vital role in the health and development of young children [1,2]. Children living in low-income communities are more likely to exhibit behavioral challenges within ECE programs [3]. Preschool children who exhibit challenging behaviors are suspended and expelled from ECE programs at three times the rate of children in K-12 schools [4], and rates are disproportionately higher for males who are ethnic minorities [5]. Research suggests that Infant and Early Childhood Mental Health Consultation (IECMHC) is one way to address challenging behaviors before they get to the point where children are expelled from preschool [6]. Yet, there are limited resources and a lack of a mental health consultation infrastructure within ECE centers to support this need [7].

To address challenging behaviors, ECE teachers need tools and skills that support the development of prosocial behaviors. Skilled teachers, working in partnership with families, play a central role in building children’s social-emotional competencies and shaping long-term developmental outcomes. IECMHC provides ECE teachers with consultation geared towards helping them identify attitudes, beliefs, practices, and conditions that promote a quality classroom environment that supports prosocial skills. The Jump Start (JS) Early Childhood Consultation is a program that uses IECMHC [8]. The program emphasizes collaboration and equal partnership with early learning program directors, recognizing their expertise and leadership in supporting young children. The JS program aims to increase the capacity of all ECE providers (i.e., family childcare homes and childcare centers) to address young children’s needs, reduce challenging behaviors that increase risk for preschool expulsion, and alleviate teachers’ job-related stress, ultimately creating a positive impact for young children [7]. Traditionally, the program uses a toolkit to provide centers with psychosocial coping strategies to boost resilience. The toolkit is organized around four program pillars: two are psychosocial (Self-Care, Trauma-Informed Behavior Support) and two are safety-related (Safety, Communication). All materials are in English and Spanish to support the linguistic needs of Miami-Dade County. Despite the existence of effective resources such as JS IECMHC, teachers often struggle to access or implement them due to staffing shortages, time constraints, or lack of awareness of available services [9,10,11]. A scalable, multi-level strategy that is easily accessible is urgently needed to support teachers in addressing child behavior challenges.

Mobile app technology has become very prevalent over the years and may increase the uptake of IECMHC practices by offering just-in-time, on-demand learning [12]. Most existing child development apps target K-12 populations, prioritize behavioral treatment over preventive efforts, and focus on a single level of intervention, such as the caregiver [13,14,15]. However, mobile apps are also becoming an increasingly common mechanism for providing support to teachers and staff in ECE programs [16]. For example, one study reviewed 43 apps designed for children’s health across iOS and Google Play. Researchers found that these apps often aimed to support chronic illness management, foster age-appropriate engagement, and ensure digital health safety. Their study highlighted the importance of designing apps for dual use by children and caregivers, aligning with the broader goals of empowering families and improving access to health interventions [17]. Another study investigated the use of a functional behavior support app with teachers who work with children exhibiting challenging behaviors by helping them develop a behavior support plan. At baseline, teachers showed infrequent use of support strategies with both their target students and the broader classroom. Following the development of behavior support plans via the app, results showed some teachers demonstrated immediate increases in strategy use, while others showed more variable implementation patterns [18].

Despite the promise of digital tools, no existing apps provide real-time, culturally responsive mental health consultation tailored to the daily realities of ECE teachers, particularly in under-resourced, linguistically diverse settings. To address this gap, this project represents the first known effort to translate a gold-standard, community-based IECMHC program into a bilingual, AI-enabled mobile app tailored for ECE teachers [19,20]. Building on the success of the in-person JS model and in response to an urgent need for accessible, low-burden support tools, Jump Start on the Go (JS Go) was developed. The JS Go app extends the program’s reach by delivering evidence-based content in a digital format with an Artificial Intelligence (AI) chatbot feature. While traditional IECMHC models are limited by consultant availability, JS Go offers 24/7 access to dynamic, evidence-based content through an integrated platform combining AI, videos, and infographics. AI serves as a digital assistant, delivering real-time, tailored guidance to teachers based on classroom dynamics, reducing intervention delays from days to seconds. JS Go reimagines public-health approaches to mental health consultation by creating a scalable, sustainable model that could transform national access to critical child mental health supports with reduced staffing of traditional approaches.

The purpose of this study was to assess the efficacy, feasibility, and utility of the JS Go mental health intervention delivered via a mobile application as compared to the control group, Jump Start traditional in-person delivery. It was hypothesized that JS Go would be as effective as the traditional Jump Start program. Specifically, the evaluation aimed to answer the following questions: Does the JS Go mobile application improve child psychosocial outcomes, reduce child challenging behaviors, and enhance teacher well-being and classroom practices, in line with the traditional Jump Start in-person consultation model? Does JS Go enhance teacher well-being and classroom practices, in line with the traditional Jump Start in-person consultation model? Was JS Go a useful and acceptable consultation mechanism, and how well was the platform received by teachers? Feasibility was examined through qualitative interviews with directors and teachers to identify implementation barriers and facilitators in real-world ECE settings. JS Go efficacy was evaluated by comparing teacher and child outcomes between the intervention and the standard Jump Start center. Together, these research questions provide a comprehensive examination of a novel mobile application approach to delivering early childhood mental health consultation in real-world ECE settings.

## 2. Materials and Methods

### 2.1. Participants

The University of Miami’s Institutional Review Board approved this study (IRB study number 20231026), and it is currently registered with ClinicalTrials.gov (NCT06374550). Participants were recruited through the ECE programs, and this included ECE directors and teachers, and parent-child dyads. Six ECEs were randomly selected from a pool of 28 ECEs that met the following criteria: (1) have >30 children ages 2-to-5; (2) serve low-income families; (2) reflect the ethnic diversity of the Miami-Dade County Public School System (60% Hispanic, 20% Non-Hispanic Black, 20% Non-Hispanic White); and (3) have teachers who agree to participate. Parents, teachers, and directors were recruited within the ECEs that were enrolled in this study. To be included, parents, teachers, and directors had to meet the following criteria: (1) must speak English, Spanish, or Creole; (2) must have a child enrolled in the ECE that is participating in this study; and (3) children ages 2–5. The exclusion criteria consisted of any participants who did not speak English, Spanish, or Creole. Participants who agreed to participate completed the informed consent process, whereas the parents/legal guardians completed the informed consent for children enrolled in this study. In accordance with ethical research practices and as per the University of Miami (UM) Institutional Review Board (IRB) requirements, study participation required caregivers to provide written consent. No children were included who would require assent, given that they were of preschool age.

### 2.2. Procedures

This was a mixed-methods, quasi-experimental matched-controlled study to assess the efficacy of the AI-enhanced digital IECMHC plus human consultation model, JS Go, relative to the traditional JS model (human consultation only). The consultation model was assigned at the childcare center level. Therefore, teachers and children either received the JS Go or the JS intervention at their childcare center. Participants were assessed at pre-intervention/baseline and at post-intervention. Participants received $20 for the baseline assessments and $30 for the post assessments. Teachers completed assessments about themselves and their children, while parents completed assessments about the sociodemographic characteristics of their children. Teachers completed the measures via the JS Go application, which has an applied programming interface with Research Electronic Data Capture (REDCap) [21]. Parents completed the measures via REDCap through email and public hyperlinks. All data were stored in the REDCap system, a secure, web-based application designed exclusively to support data capture for research studies.

Matching for the current study occurred at the child level. Children enrolled in JS Go were matched with children in a previously conducted JS research trial [22]. Children in the JS group (*n* = 87) were first excluded from the matching process if they were missing both baseline teacher-reported assessments: (1) the Devereux Early Childhood Assessment (DECA) and (2) the Strengths and Difficulties Questionnaire (SDQ) (*n* = 14). Of the remaining children, an additional 16 were excluded for missing either the teacher-reported DECA or SDQ at baseline. This resulted in a final JS sample of 57 children, which aligned with the JS Go sample (*n* = 57).

Beyond quantitative measures, directors (*n* = 3) and teachers (*n* = 4) within the JS Go intervention completed semi-structured interviews immediately following the JS Go intervention. An interview guide was developed (see Table 1) based on four domains of the RE-AIM framework (effectiveness, adoption, implementation, and maintenance) to explore the center staff’s experience with the JS Go app [23]. The same interview guide was used with both directors and teachers. Examples of questions included, “Tell me about your experience with the JS Go program,” and “What do you need to maintain the JS Go program, specifically using the app on a regular basis, and use of the strategies in your center?” The bilingual mental health consultant (MHC) who implemented the intervention and was previously trained on rapid qualitative analysis (RQA) conducted interviews in participants’ preferred language using the Zoom videoconferencing platform. Interviews lasted on average M  =  31.9 min, SD  =  11.6 min. All interviews were recorded via Zoom and securely transferred to a HIPAA-compliant university cloud storage system. The MHC downloaded and reviewed the audio files generated by Zoom to ensure accuracy. All interviews were conducted in Spanish and transcribed by the MHC using Microsoft Word Online’s transcription feature. All transcripts were analyzed in their original language.

#### 2.2.1. JS Go Intervention

JS Go is an early childhood mental health consultation program that utilizes a hybrid model combining an AI-enhanced mobile application with live mental health consultation. The program is similarly built upon Georgetown University’s evidence-based Infant/Early Childhood Mental Health Consultation (I/ECMHC) framework and encompasses the same four fundamental pillars as the traditional JS model: (1) Safety, (2) Behavior Support, (3) Self-Care, and (4) Communication [7,24].

The JS Go model implements a multi-level, multi-modal intervention that provides (1) simplified multilingual video content in English and Spanish within an app, (2) personalized AI-driven guidance aligned with the JS Go curriculum, and (3) secure two-way communication between teachers and consultants. The program was designed through a community-based participatory research approach, ensuring cultural and linguistic relevance for diverse childcare center settings [20].

The JS Go intervention was implemented over 14 weeks, to match the duration of the JS intervention. Participants received weekly one-hour live consultation sessions with mental health consultants focusing on the four core pillars. In addition, the consultant modeled how the teacher could utilize the JS Go app for ongoing support, education, and AI-driven guidance between consultation sessions. The JS Go app includes three key components: multimedia modules demonstrating implementation strategies, AI-augmented support providing on-demand personalized guidance with safety protocols that escalate complex needs to human consultants, and connected communication allowing secure messaging between teachers and consultants with responses within one business day. The AI augmented support chatbot of JS Go was developed and trained as a retrieval-based model in which teachers input questions or describe situations, the system matches these inputs against a database of pre-approved responses, examples, and guidance from the Jump Start curriculum. The AI system implements programmed boundaries that prevent responses outside of the JS Go scope, particularly for crisis-level situations, and withholds responses when confidence thresholds for accuracy are not met. Specifically, the AI includes safety mechanisms that automatically escalate complex cases to human consultants, maintaining expert oversight for critical situations.

This study consisted of implementing a toolkit which included (1) infographics with key points, a reflective question, and applicable tips that pertain to intervention pillars (Self-Care, Behavior Support, Safety, and Communication); and (2) how-to videos demonstrating strategies for each pillar, such as resiliency-based coping ideas for teachers and directors. Structure: The Action Plan is a standardized implementation protocol used by the MHC in each consultation to (1) set the goal based on the pillar for the week, (2) provide strategies for achieving the goals using infographics and “how to” videos located on the JS Go app, (3) have teachers practice strategies while the consultant provides real-time feedback. Approximately three weeks are devoted to each pillar before moving on to the next. The order of pillar discussion was based on a tailored approach considering the areas of need/weaknesses from each participant’s Health Environment Rating Scale (HERS) self-assessment (measure discussed later) as well as a conversation with each participant on their agreement with the proposed plan. Dose: the MHC provided 14 weeks of in-person consultations for 1.5 h weekly per center. Classroom-level consultations occurred individually and/or in a small group (two teachers) for 30 min per week, during nap time at the center, to provide teachers with goals/strategies to adopt pillars into their daily lives and manage challenging behaviors of specific children in the classroom. Examples of goals/strategies include helping teachers develop a self-care plan to build resiliency (beliefs) and use coping strategies (self-efficacy). Reflective consultation was facilitated with the use of infographics and videos during the live sessions. For example, reflective questions were asked about the infographics, and both videos and infographics were used to start discussions about pillars and goals. Teachers were shown examples of how to access and utilize the app and were assigned homework between consultations to utilize the chat, review infographics, and watch videos.

**Figure 1:**
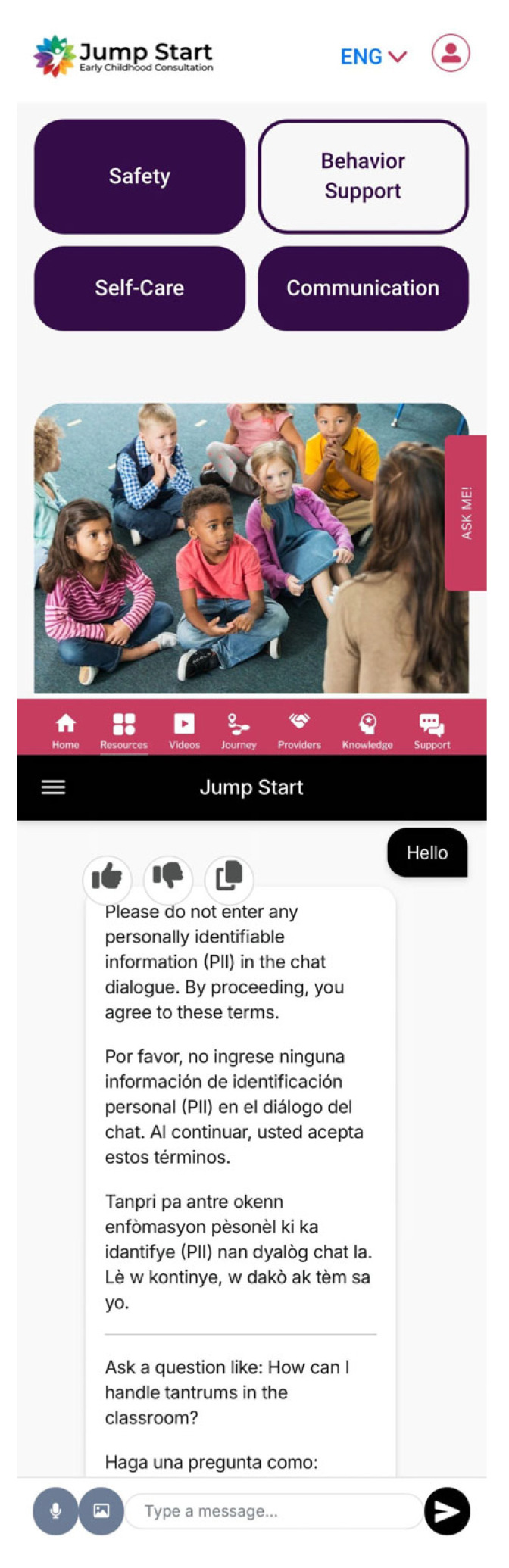
JS Go User Interface.

The JS Go app served as the primary platform for accessing all toolkit components, including infographics, videos, and an AI-powered chatbot called Avatar Buddy (see Figure 1). The app is a multilingual tool (available in English, Spanish, and Creole) designed to provide IECMHC resources and evidence-based strategies aimed at reducing suspensions and expulsions in early childhood education programs. Key app sections include (a) the **Home** page for central navigation; (b) the **Resources** page, which houses 16 infographics on IECMHC topics such as expulsions and suspensions, discipline, emergency planning, etc. App users can apply specific filters to access content based on their preferred ECE setting level (program/center, classroom, or child) and/or the JS pillars (safety, behavior support, self-care, and communication); (c) the **Videos** page, which contains brief educational videos aligned with infographic topics and filtered using the same logic; (d) the **Journey** page, which serves as the secure login hub for survey completion; (e) the **Providers** page, offering information about JS community partners; and (f) the **Support** page, which features the AI chatbot equipped to answer questions related to IECMHC topics in both English and Spanish.

#### 2.2.2. Matched–Control. Traditional Jump Start

The JS program is an early childhood mental health consultation initiative modeled after Georgetown University’s evidence-based I/ECMHC framework. This model encompasses four fundamental pillars: (1) Safety, (2) Behavior Support, (3) Self-Care, and (4) Communication [7,24]. JS is grounded in the Caring for Our Children-National-Health and Safety Standards [25], complemented by CDC COVID-19 guidelines for childcare centers [26], and evidence-based practices for enhancing children’s social competence [27].

The consultation model was implemented through weekly one-hour sessions over a 14-week period, delivered by consultants utilizing telepresence robots. JS aimed to (1) enhance children’s social, emotional, and behavioral development, and (2) prevent and/or address behavioral challenges by strengthening the capacity of all caregivers (directors, teachers, parents/families), thereby reducing and preventing preschool suspensions and expulsions.

Each pillar of the JS model features a collection of 24 infographics (six per pillar) tailored for classroom teachers. These infographics follow a standardized format with three sections: (1) Reflect, (2) Inform, and (3) Practice. They serve as guides through the consultative process, enabling childcare staff to reflect on their practices and collaboratively develop personalized professional goals.

#### 2.2.3. Consultant Training

The JS and JS Go consultants were clinically trained early childhood mental health professionals with supervision, many holding endorsements from a state infant mental health association. Each consultant underwent extensive training in implementing the JS program, including six virtual onboarding sessions: (1) introduction to Georgetown University’s I/ECMHC/Jump Start model and its four pillars, (2) program-level observation training, (3) classroom-level observation training, (4) curriculum infographic review, and (5) exemplar consultation video analysis. JS Go consultants received additional training in how to integrate the use of the JS Go app within consultation services with teachers.

### 2.3. Measures

#### 2.3.1. Sociodemographic Characteristics

Teacher demographic characteristics (e.g., age, gender, race, ethnicity, preferred language, level of education, and years of experience in the ECE setting) were collected via the JS demographic survey at pre-intervention only. Family characteristics (i.e., age, gender, race, ethnicity, primary language spoken in the home, English proficiency) were measured by the JS demographic survey at pre-intervention only.

#### 2.3.2. Teacher Measures

The Health Environment Rating Scale-Classroom (HERS-C) is a 30-min observational assessment developed by our study investigators and used to evaluate classroom implementation across four domains: safety, behavioral supports, communication, and resiliency coping. These domains correspond to both national health and safety standards for early childhood education programs and the four JS Go pillars [8,25]. HERS-C items assess core classroom practices, such as “Classroom has safety guidelines and procedures for responding to crisis situations and adheres to CDC/DCF guidelines” and “Teacher shares behavior expectations and classroom rules using positive language, praise, and redirection when needed”. Observers rated implementation on a 7-point Likert scale ranging from “little or no implementation” (1) to “excellent implementation” (7). The measure demonstrated good internal consistency (α = 0.82) in this study, and all four domains were examined.

Teachers’ beliefs related to their job were measured by the Childcare Worker Job Stress Inventory (CWJSI), which assesses three domains of workplace stress, including job demands, resources, and control [28]. Each subscale is 17 items with responses rated from “very little” (1) to “very much” (5). Sample items include “I feel like I have to be a parent and a teacher to the children” (job demands), “I get praise from the parents for the work that I do” (job resources), and “How much control do you have over the number of children you care for?” (job control). Higher scores indicate greater perceived control, resources, and job-related demands. The domains have strong internal consistency and constructive validity and have been used effectively in prior JS research studies with low-income children [29].

The Teacher Opinion Survey assessed teachers’ confidence in managing challenging child behaviors [30]. This 12-item self-report measure uses a 5-point Likert scale from “strongly disagree” (1) to “strongly agree” (5), with higher scores indicating greater confidence. Sample items include, “If I keep trying, I can find some way to reach even the most challenging child”, and “If a student in my class became disruptive and noisy, I feel pretty sure that I would know how to respond effectively”. The total score demonstrated adequate internal consistency (α = 0.79).

The Brief Resilient Coping Scale was employed to measure teachers’ tendencies to cope adaptively with stress. This 4-item instrument, which includes items such as “I look for creative ways to alter difficult situations” and “I believe I can grow in positive ways by dealing with difficult situations”, has established reliability and validity [31]. It uses a 5-point rating scale from “does not describe me at all” (1) to “describes me very well” (5). Total scores classify respondents as low (4–13 points), medium (14–16 points), or high (17–20 points) resilient copers. The scale showed good internal consistency in this study (α = 0.835).

Consultation dosage was operationalized as the total number of consultation sessions each teacher received during the intervention period.

#### 2.3.3. Teacher Technology Acceptability of JS Go App Measures

The Technology Acceptance Model Instrument-Fast Form (FF-TAM) is a 16-item checklist that evaluates attitudes toward technology use [32]. It can be modified by listing the technology of interest. Within this study, the mobile application was listed as the technology. The FF-TAM uses an 8-point semantic differential scale ranging from −4 (e.g., inefficient) to +4 (e.g., efficient) to rate items, allowing for a broad range of responses. The FF-TAM has three subscales designed to measure different aspects of technology acceptance, including Usefulness (e.g., unhelpful vs. helpful), Ease of Use (e.g., very cumbersome vs. very usable), and Predicted Future Usage (e.g., For future consultation tasks that are totally within my control, I would probably use telepresence robots as a consultation platform.). All three subscales (i.e., usefulness, ease of use, and predicted future usage) were used in this study. Childcare staff only completed this measure immediately post-intervention. Internal consistency for each of the subscales was high for the childcare staff (α = 99 for all scales).

Participants completed the mHealth App Usability Questionnaire (MAUQ) in either English or Spanish [33]. The MAUQ is a short, reliable, and customizable questionnaire designed to assess the usability of mobile apps and gauge teachers’ perceptions of the usefulness of the JS Go app, the quality of the user interface, and the perceived usefulness of the app in delivering early childhood mental health consultation content. Data were collected post-intervention, for a total of one time point. The MAUQ has 18-items organized into three subscales: ease of use and satisfaction (e.g., “It was easy for me to learn to use the app”), system interface arrangement (e.g., “The information in the app was well organized, so I could easily find the information I needed”), and usefulness (e.g., “The app has all the functions and capabilities I expected it to have”). Participants rated JS Go using a seven-point Likert scale (1 strongly disagree to 7 strongly agree). The Cronbach’s alpha for the MAUQ in this sample was α = 0.90 for the Ease of Use subscale, α = 0.93 for the Interface subscale, and α = 0.92 for the Usefulness subscale.

In addition to the MAUQ, the Mobile Application Rating Scale (MARS) in English and Spanish was used to classify and assess the quality of mobile health apps [34]. This 23-item tool was used to measure app objective quality indicators of engagement, functionality, aesthetics, and information quality, as well as app subjective quality. The app quality scores were clustered within the engagement, functionality, aesthetics, and information quality subscales. Sample items include, “Is the app fun/entertaining to use?” (engagement), “How accurately/fast do the app features (functions) and components (buttons/menus) work?” (functionality), “How good does the app look?” (aesthetics), “Is app content correct, well written, and relevant to the goal/topic of the app?” (information quality), and “Would you recommend this app to people who might benefit from it?” (app subjective quality). Each MARS item used a 5-point scale (1-Inadequate, 2-Poor, 3-Acceptable, 4-Good, 5-Excellent). In cases where an item may not be applicable for all apps, an option of Not applicable was included. Data were collected post-intervention, for a total of one time point. The Cronbach’s alpha for the MARS in this sample was α = 0.96 for the functionality subscale, α = 0.90 for the aesthetics subscale, and α = 0.93 for the information quality subscale.

#### 2.3.4. Child Measures

The Devereux Early Childhood Assessment (DECA) instrument was used to measure protective factors that promote resilience in young children. These included the DECA for Infants and Toddlers (DECA-I/T) [35] and the DECA for Preschoolers, Second Edition (DECA-P2) [36], both validated, reliable, standardized, and norm-referenced teacher-report measures for children from 1 month through 5 years [37,38]. The DECA-I/T consists of 36 items, while the DECA-P2 contains 38 items, each rated on a five-point scale from “never” (0) to “very frequently” (4). Both instruments yield three subscales: Initiative (ability to use independent thoughts and actions to meet needs), Self-Regulation (ability to express emotions and behaviors in healthy ways), and Attachment/Relationships (mutual, strong relationships between children and significant adults), as well as a Total Protective Factors (TPF) score. Each DECA item begins with the prompt “During the past 4 weeks, how often did the toddler/child…” followed by behaviors such as “show affection for familiar adults” and “calm herself/himself down”. These measures have demonstrated adequate internal consistency in English and Spanish-speaking, low-income, diverse samples [39]. In the current study, the internal consistency for the DECA total protective factors scale was excellent (α = 0.975).

The Strengths and Difficulties Questionnaire (SDQ) was used to assess behavioral problems in children. This 25-item screening measure is designed for youth ages 2–17, with specific versions for 2–4-year-olds and 4–17-year-olds, though it has also shown promising reliability for 12–24-month-old children (with better reliability for externalizing than internalizing subscales). The teacher-report version was utilized in this study, with items rated on a three-point Likert scale: “not true” (0), “somewhat true” (1), and “certainly true” (2). Each item describes a specific child behavior, such as “Considerate of other people’s feelings”, “Often loses temper”, and “Many worries or often seems worried”. The SDQ comprises five domains: emotional symptoms, conduct problems, hyperactivity/inattention, peer relationship problems, and prosocial behavior. An externalizing score was calculated by summing the conduct problems and hyperactivity/inattention scales, while an internalizing score combined the emotional and peer problems scales. The SDQ has established validity and reliability [40], and in this study, the internal consistency for the total problems scale was good (α = 0.860).

### 2.4. Data Analysis

#### 2.4.1. Quantitative Analysis

Baseline demographic characteristics of teachers and children were summarized by intervention group (JS Go and JS) using descriptive statistics. Categorical variables were reported as frequencies and percentages, and continuous variables were summarized using means and standard deviations (SD). Between-group comparisons for categorical variables were conducted using Chi-square tests or Fisher’s exact tests, as appropriate. For continuous variables, independent sample *t*-tests or ANOVA were used depending on the distribution of the data.

For the evaluation of teacher outcomes, mean scores and standard errors (SE) at baseline (T0) and follow-up (T1) were calculated for each outcome by treatment group. Change scores (T1–T0) were also calculated to describe within-group changes over time. To formally test intervention effects, analysis of covariance (ANCOVA) models were conducted for each outcome, with T1 scores as the dependent variable, intervention group (JS Go vs. JS) as the independent variable, and T0 scores as covariates to adjust for baseline differences. The JS group was used as the reference group. Models were estimated using PROC SURVEYREG in SAS, specifying the program as the clustering variable to account for nesting of teachers within programs and to produce robust standard errors. Model coefficients (β), standard errors, and *p*-values were reported for the effect of treatment group.

To examine changes in child outcomes over time and between treatment groups, generalized estimating equation (GEE) models with an exchangeable correlation structure were used to account for clustering of repeated measurements within participants and clustering within programs. For each outcome, models included main effects for time (T0 vs. T1), treatment group (JS Go vs. JS), and their interaction (time × treatment), with JS and baseline (T0) as reference categories. The models assumed a normal distribution and identity link function. Least square means (LS-means) were calculated to estimate and compare group means over time. A two-sided *p*-value of <0.05 was considered statistically significant. As a sensitivity analysis, all GEE models were re-estimated with additional adjustment for child primary language (Spanish vs. English) to evaluate whether intervention effects were consistent across language groups. The models included main effects of child language and treatment × time × language interactions. A two-sided *p*-value of <0.05 was considered statistically significant. All analyses were conducted using SAS 9.4 and visualized using R (version 4.4.2) [41,42].

#### 2.4.2. Qualitative Analysis

Rapid Qualitative Analysis (RQA) was used to identify themes from semi-structured interviews with seven JS Go center staff (three directors and four teachers) [43,44,45,46,47]. RQA is a validated, time-efficient method grounded in implementation science and designed to maintain methodological rigor while producing timely findings [46]. To guide interviews, a semi-structured protocol was developed by the research team based on implementation science frameworks and the core components of the Jump Start model. The protocol was reviewed for content validity by two IECMHC experts and piloted with a non-study teacher to ensure clarity and cultural responsiveness.

Interview transcripts were first summarized using a standardized template developed by the research team. These templates captured key points across predefined domains (e.g., app use, challenges, perceptions of impact, and recommendations). The MHC who conducted the interview prepared the initial summaries immediately after each session. A second trained research assistant then extracted salient content from the summaries and transferred it into a structured analytic matrix. This matrix allowed for rapid comparison of key themes across participants.

A third team member conducted thematic analysis using the matrix to identify recurring concepts. These initial themes were refined through iterative discussions with the broader study team, including investigators with qualitative and IECMHC expertise. Inter-coder reliability was supported through team-based triangulation and consensus-building discussions. An audit trail was maintained throughout the analytic process to ensure dependability and confirmability of findings.

## 3. Results

### 3.1. Teacher Outcomes

#### 3.1.1. Teacher Characteristics

A total of 28 teachers participated in the matched controlled comparison, including 6 in the JS Go group and 22 in the JS group (See Table 2). Given that groups were matched on child characteristics, there was an unequal distribution of total teachers across groups. All participants identified as female and Hispanic, and reported Spanish as their primary language. There were no significant differences between groups in terms of teacher age, race, education, years of experience, consultation dosage, and/or intervention completion.

#### 3.1.2. Teacher Descriptive Outcomes at Baseline and Follow-Up

Descriptive statistics for each outcome at baseline and follow-up are summarized in Table 3 and visually shown in Figure 1. At pre-treatment, scores were generally similar across groups for most outcomes. JS Go teachers showed increases from baseline to follow-up in several domains, including HERS-C Safety (+0.42), HERS-C Behavior (+0.45), HERS-C Communication (+0.30), and HERS-C Resiliency (+1.27). JS participants also demonstrated improvements in HERS-C Safety (+0.31), HERS-C Communication (+0.07), and HERS-C Resiliency (+0.43).

In contrast, JS Go participants had decreases in Brief Resiliency Coping (−1.33) and Teacher Opinion Survey scores (−1.83), while JS participants had smaller changes (−0.19 and +1.03, respectively). For Childcare Worker Job Demands, JS Go showed little change (−0.04), whereas JS participants increased by +0.81 points, indicating higher demands. Patterns for Childcare Worker Job Resources and Childcare Worker Job Control indicated slight declines for both groups over time.

#### 3.1.3. Teacher Intervention Effects from ANCOVA Models

Results from ANCOVA models evaluating intervention effects at post-treatment (T1), adjusting for pre-treatment values (T0), revealed significant differences between the JS Go and JS groups for several outcomes (See Table 4). All models were adjusted for clustering by program (*n* = 6), and robust standard errors were estimated with degrees of freedom based on the number of clusters (df = 5), ensuring that variability between programs was appropriately accounted for in the inference. Specifically, JS Go participants had significantly lower scores on Childcare Worker Job Demands (β = −0.60, SE = 0.12, *p* = 0.0041), indicating lower perceived job demands relative to JS. JS Go participants also had significantly higher scores on HERS-C Behavior (β = 0.33, SE = 0.13, *p* = 0.0474), suggesting that teachers engaged in a higher rate of effective classroom practices to support adaptive child behavior within the classroom. Trend-level differences were observed for HERS-C Safety (β = −0.39, SE = 0.15, *p* = 0.0506), HERS-C Communication (β = −0.49, SE = 0.21, *p* = 0.07), and HERS-C Resiliency (β = 0.68, SE = 0.32, *p* = 0.0876), with JS Go teachers reporting fewer safety and communication classroom practices but more resiliency practices compared to JS teachers. No significant between-group teacher differences were found for Brief Resiliency Coping, Teacher Opinion Survey, Childcare Worker Job Resources, or Childcare Worker Job Control.

### 3.2. Child Outcomes

#### 3.2.1. Child Characteristics

Table 5 presents the baseline demographic characteristics of the child sample across groups (*N* = 114). There were no significant differences between treatment groups on age, gender, ethnicity, or English proficiency. However, there was a significant difference in racial composition (*p* = 0.023), with a higher proportion of Black children in the JS group (8%) compared to JS Go (0%). The majority of participants identified as White and Hispanic across both groups.

#### 3.2.2. Descriptive Statistics of Child Outcomes

Table 6 presents the means, standard errors, and change scores of child outcomes at baseline and follow-up by treatment group. At baseline, children in the JS Go group had lower scores on DECA subscales, including Attachment (44.04 [SE = 1.39]), Initiative (46.61 [SE = 1.22]), Self-Regulation (50.53 [SE = 1.47]), and Total (46.81 [SE = 1.39]), and higher scores on SDQ subscales, including Externalizing (6.79 [SE = 0.49]), Internalizing (5.30 [SE = 0.54]), and Total (9.54 [SE = 0.76]), relative to JS participants (DECA Attachment: 47.77 [SE = 1.10]; Initiative: 50.98 [SE = 1.33]; Self-Regulation: 52.68 [SE = 1.29]; Total: 50.81 [SE = 1.25]; SDQ Externalizing: 4.53 [SE = 0.60]; Internalizing: 4.14 [SE = 0.58]; Total: 6.93 [SE = 0.89]). These differences suggest that JS Go participants had a less favorable starting point compared to JS in terms of protective factors and behavioral functioning.

**Figure 2:**
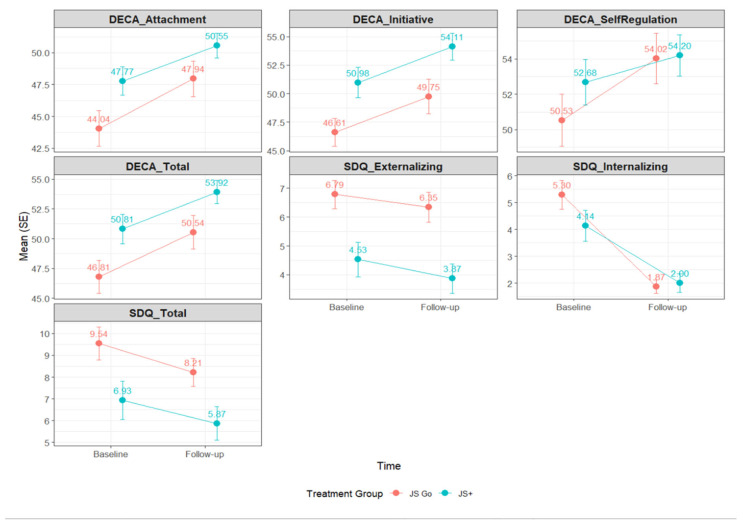
Teacher-reported improvements in child outcomes over time by intervention group.

Both groups demonstrated improvements from baseline to follow-up (See Figure 2), though the magnitude of change varied across outcomes. For DECA subscales, JS Go participants improved by 3.91 points in Attachment (follow-up M = 47.94 [SE = 1.39]), 3.14 points in Initiative (49.75 [SE = 1.51]), 3.49 points in Self-Regulation (54.02 [SE = 1.42]), and 3.73 points in Total (50.54 [SE = 1.42]). JS participants showed comparable improvements with increases of 2.77, 3.13, 1.52, and 3.12 points in Attachment (50.55 [SE = 0.96]), Initiative (54.11 [SE = 1.18]), Self-Regulation (54.20 [SE = 1.16]), and Total (53.92 [SE = 0.99]), respectively.

Similarly, reductions in SDQ scores were observed, reflecting fewer behavioral concerns over time. Among JS Go participants, Externalizing scores decreased by 0.44 points to 6.35 (SE = 0.52), Internalizing decreased by 3.43 points to 1.87 (SE = 0.25), and Total scores decreased by 1.33 points to 8.21 (SE = 0.64). In JS, reductions of 0.65, 2.14, and 1.06 points were observed for child Externalizing (3.87 [SE = 0.51]), Internalizing (2.00 [SE = 0.34]), and Total (5.87 [SE = 0.78]) scores, respectively.

#### 3.2.3. GEE Analysis of Child Outcomes

Results of the updated GEE models are shown in Table 7. Significant main effects of time were observed for DECA Attachment (β = 2.89, *p* = 0.023), DECA Initiative (β = 3.05, *p* = 0.011), DECA Total (β = 3.22, *p* = 0.008), and SDQ Internalizing (β = −2.18, *p* < 0.001), indicating meaningful within-person changes over time regardless of treatment group.

Significant main effects of treatment group (JS Go vs. JS) were found for DECA Attachment (β = −3.74, *p* = 0.034), DECA Initiative (β = −4.37, *p* = 0.015), DECA Total (β = −4.00, *p* = 0.031), SDQ Externalizing (β = 2.26, *p* = 0.003), and SDQ Total (β = 2.61, *p* = 0.024), indicating that JS Go participants had lower DECA scores and higher SDQ scores compared to JS participants when averaged across time points. No significant time × treatment interactions were detected for any outcomes, indicating that the degree of improvement from baseline to follow-up did not significantly differ by treatment group.

In the sensitivity analysis, adjusting for child primary language, intake of child language (Spanish vs. English) was not significantly associated with any outcomes. Across all models, *p*-values for language were >0.10, suggesting no meaningful differences in change or post-intervention levels between Spanish-speaking and English-speaking children. No treatment × language interactions were detected, indicating that intervention effects were generally consistent across language groups.

### 3.3. Teacher Acceptability Ratings of JS Go App

Descriptive statistics for technology acceptability and app quality are presented in Table 8. Overall JS Go teachers reported high levels of perceived usefulness (M = 3.56, SD = 1.01) and ease of use (M = 3.67, SD = 0.82) of the JS Go app on the Technology Fast Form (TFF), though predicted future usage showed greater variability (M = 1.38, SD = 3.63). On the mHealth App Usability Questionnaire (MAUQ), participants rated the app highly across all domains, with average scores near the upper limit of the 7-point scale for ease of use (M = 6.43, SD = 0.59), user interface (M = 6.52, SD = 0.46), and usefulness (M = 6.47, SD = 0.45). Ratings from the Mobile Application Rating Scale (MARS) indicated that teachers found the JS Go app to be of high quality across all subdomains, including engagement (M = 4.73, SD = 0.24), functionality (M = 4.79, SD = 0.51), aesthetics (M = 4.72, SD = 0.53), and information quality (M = 4.67, SD = 0.42). The overall subjective app quality rating was also high (M = 4.73, SD = 0.38), suggesting strong user satisfaction.

### 3.4. Rapid Qualitative Analysis of Staff Perceptions of JS Go App Feasibility

Interview participants (three directors and four teachers from JS Go centers) identified as White Hispanic females, with a mean age of 50.63 (SD = 9.76) years and 15.0 (SD = 9.58) years of ECE experience. All reported Spanish as their primary language, and 43% had earned a high school diploma or GED as their highest level of education. Semi-structured interviews yielded five primary themes reflecting the center staff’s experiences with the JS Go intervention. Specifically, these included (1) the app as a practical and motivating tool with accessibility gaps, (2) the MHC’s role in facilitating reflective practices, (3) staff self-efficacy in implementing program pillars, (4) opportunities to strengthen parent engagement, and (5) organizational supports and incentives needed for program sustainability. Themes and translated illustrative quotes are presented in the text below.

*Theme 1: JS Go App as a Practical and Motivating Tool with Accessibility Gaps*

Staff consistently described the JS Go app as a well-organized and user-friendly resource that supported regular classroom implementation of program pillars. The app’s clearly structured content, instructional videos, and AI chat feature were widely praised for offering practical, just-in-time support in reinforcing key strategies and responding to children’s challenging behaviors. One teacher noted, “The videos have been a tremendous help in strengthening my experience as a teacher. They have helped me a lot, with routines and with improving how I respond to children’s disruptive behaviors”. Another, referring to the AI chatbot feature, added, “If you have a direct question, you get an answer along with examples. I like it because it does not just give you the answer, it gives you several examples of how to develop what you are asking about”. Participants noted that they frequently revisited the app to access its repository of resources when navigating difficult classroom situations. However, the app’s browser-only format was identified as a barrier to consistent access. Participants expressed a strong preference for a downloadable version available through the app store that could function offline, especially in centers with unreliable internet access. As one teacher explained:

I would like it to be an app that can be downloaded and that offers more resources. Resources that can be taught directly to the children, things we can incorporate into the lesson plan, or in circle time. Having the app be more interactive and easier to have on hand on my phone.

*Theme 2: The MHC’s Role in Facilitating Reflective Practices*

In-person consultation emerged as an essential program component that provided irreplaceable human connection and individualized support. Participants described these interactions as creating reflective spaces where they could process challenges and receive emotional validation. One director noted, “…it is like having a private psychologist. I really enjoyed it because, as I listened to myself, I was self-analyzing my responses and reactions at that moment. That helped me and made me think differently for future occasions”. The MHC was consistently viewed as someone who offered both emotional support and practical guidance, particularly in areas such as self-care, racial equity, and communication with families. While participants appreciated the app’s streamlined format and usefulness, some emphasized the importance of real dialogue and idea exchange with the consultant. As one teacher explained, “With the app you go straight for the question or you watch a video, but when you are talking, you know, like the communication, it is more. You can share ideas, and sometimes we need to talk”. This consultation component, combined with the app’s resources, facilitated professional growth by reinforcing existing practices and introducing new strategies. Participants emphasized that the consultant’s approach made complex or sensitive topics more approachable and helped build trust and confidence among staff.

*Theme 3: Staff Self-Efficacy in Implementing Program Pillars*

Participants described increased confidence in applying key components of the JS Go program, particularly in managing classroom behaviors, supporting their own and children’s emotional regulation, and promoting a sense of safety. For instance, one director described a shift in her understanding of safety to include not only physical protections but also children’s emotional well-being, stating, “It is not just about structure or the physical aspect; it is something more as well”.

Additionally, the toolkit materials were frequently cited as helpful for reinforcing strategies introduced in the app and during consultation. Several participants described feeling more capable of responding to challenging behaviors through preventive strategies, positive reinforcement, and relaxation techniques (e.g., verbal reminders, role modeling, “flowers for breathing” method). They noted that these approaches were becoming part of their regular classroom routines, helping them respond more calmly and consistently. One teacher shared, “I feel like I am doing my best and doing things right because I have seen results in the children, and others have told me so too”.

*Theme 4: Opportunities to Strengthen Parent Involvement*

Despite JS Go’s emphasis on supporting children’s development across settings, parent engagement remained limited throughout implementation. Most participants described minimal communication with families about the program beyond survey distribution or brief, informal updates. While some staff reported mentioning aspects of the program to a few parents, there were no structured strategies or built-in mechanisms for engaging families in the app or reinforcing content at home. Participants acknowledged this as a missed opportunity and expressed interest in strengthening the family engagement component, particularly through more concrete tools or guidance on how to involve parents more intentionally. One director suggested, “That access, and maybe a consultation, or maybe some kind of pages where parents could do the same things, we should definitely incorporate parents more, because they really do need it”. Another director noted the potential for the AI chatbot to support families without judgment, stating:

I think it would be a kind of support for them because they can consult any doubts, and it is like when you consult artificial intelligence. It gives you that immediate response, with a database that is exactly related to what we are looking for with children. So yes, I think it would be a support because sometimes parents might feel like, “Oh, I am not doing a good job as a mom or dad”, but they do not want to say it out loud. So, the app feels more private, and they can consult it.

*Theme 5: Organizational Supports and Incentives Needed to Sustain JS Go*

While participants expressed interest in sustaining the JS Go program, they emphasized that ongoing implementation would depend on both structural supports and staff incentives. Directors and teachers highlighted the importance of leadership involvement, adequate staffing, and time for planning and reflection. Embedding program strategies into existing structures, such as team meetings or onboarding processes for new center staff, was also seen as critical for maintaining continuity. In addition to these structural supports, several participants pointed to the opportunity to earn “training hours” and endorsed the inclusion of interactive components (e.g., “gaming features”, “unlocking digital rewards”) as factors that could help maintain participation and enthusiasm over time. Regular app updates, including new courses or training announcements, were also viewed as important for sustaining center staff engagement, given the lack of up-to-date content in many programs and teachers’ ongoing need to earn in-service hours.

## 4. Discussion

The current study used a mixed methods approach to examine the feasibility, efficacy, and utility of the JS Go mobile application as a mechanism for delivering IECMHC to early childcare centers. This novel approach integrated traditional in-person consultation with an AI-enhanced mobile platform to address a critical need for accessible mental health support in early childhood settings, particularly those serving diverse, low-income communities. Overall, the findings suggest that JS Go represents a promising digital adaptation of the established Jump Start consultation model, with several important implications for future practice and research.

One objective of this study was to determine if JS Go enhances teacher well-being and classroom practices, in line with the traditional Jump Start in-person consultation model. Teacher outcomes demonstrated some promising trends in favor of the JS Go intervention. Most notably, teachers in the JS Go group reported significantly lower job demands compared to those in the traditional JS group, suggesting that the mobile application may have served to alleviate some of the occupational burden associated with managing challenging behaviors. Further, classroom observation data revealed that JS Go teachers demonstrated significantly higher implementation of effective behavior support practices compared to the traditional JS group. This suggests that the on-demand, accessible nature of the application may have facilitated greater uptake of evidence-based strategies within childcare classrooms. This is especially notable given that 87% of teachers in previous JS research reported feeling overwhelmed by classroom behavioral challenges [48]. The app’s multilingual features, infographics, and AI-powered guidance may have provided “just-in-time” learning opportunities that not only reinforced the consultation content between sessions but also may have facilitated more consistent use of behavior support strategies. In terms of other teacher outcomes, JS Go and JS teachers demonstrated similar increases in their observed implementation of the other JS pillars (Safety, Communication, and Resiliency) over time. There were no differences between groups in terms of teacher resilient coping and teacher perceptions of job control and resources. However, it is possible that, given the small sample size for teacher groups (JS Go *n* = 6 teachers; JS *n* = 22 teachers), this study was underpowered to detect significant changes at the teacher level.

Another objective of this study was to determine whether the JS Go mobile application improves child psychosocial outcomes and reduces child challenging behaviors. Children in both the JS Go and JS groups showed significant improvements over time across multiple domains of psychosocial functioning, including teacher perceptions of children’s attachment, initiative, and reductions in internalizing behaviors. These positive trajectories align with previous IECMHC research demonstrating benefits for child outcomes [7,49] and provide preliminary evidence that the AI-hybrid digital adaptation of Jump Start maintains effectiveness for child development. If further research confirms equivalent effectiveness, the JS Go digital platform could serve as a scalable and sustainable model for expanding access to early childhood mental health consultation services. Given that fewer than 10% of childcare centers regularly implement IECMHC due to significant workforce and resource constraints [50], JS Go may help extend preventive, multilevel supports to underserved preschool-aged children.

Researchers were also interested in evaluating the utility of JS Go as a consultation mechanism by analyzing teacher ratings of the app’s usability and technology acceptability, to assess how well the platform was received and its practical value for teacher end users. The high acceptability ratings from teachers provide compelling evidence for the feasibility of integrating mobile technology and AI support into early childhood mental health consultation. Teachers rated the JS Go app favorably, with particularly strong views of the ease of use, functionality, and information quality. This provides preliminary support that the JS Go app effectively translated the complex content of IECMHC into an accessible digital format that met teachers’ needs and expectations. While there was more variability in teachers’ perception of their predicted future usage of the app, all other domains were rated highly, suggesting that with appropriate implementation support by mental health consultants, teachers may be more likely to continue to engage with the digital platform over time.

The multilingual design of JS Go appears to have been well-received by this predominantly Spanish-speaking sample, addressing an important gap in culturally and linguistically responsive resources for IECMHC. Of note, there were no differences in child outcomes regardless of whether the child preferred to speak English or Spanish. This feature likely contributed to the high usability ratings and underscores the importance of co-developing with the community, including digital tools that reflect the diversity of the early childhood workforce.

Findings from the RQA illustrate the complementary roles of technology and in-person support and highlight both implementation successes and areas of growth. For future program development, four key recommendations emerge: (1) improve app accessibility through a downloadable version; (2) strengthen the family engagement component with concrete strategies for parent involvement; (3) maintain the dual approach of technology and personal consultation; and (4) develop mechanisms for regular content updates and ongoing training to sustain engagement and implementation over time. The JS Go app emerged as a practical, on-demand resource that helped center directors and teachers reinforce key strategies related to behavior support, self-regulation, and emotional safety. Center staff found the app especially useful for reinforcing practices introduced during consultations, revisiting strategies as needed, accessing resources on their own time, and using the AI chatbot to receive quick, example-based guidance. When paired with individualized consultation, it appeared to facilitate the integration of program pillars into teachers’ daily classroom routines, with teachers expressing increased confidence in implementing JS Go practices. To further enhance engagement and implementation sustainability, a token-based gaming feature is being developed that tests users’ knowledge of the four program pillars and provides visual tracking of progress. These targeted enhancements are designed to promote staff self-efficacy, implementation fidelity, and program sustainability across diverse ECE settings. Overall, these findings offer practical guidance for the continued development of digital tools that are both scalable and responsive to the realities of ECE programs.

### Limitations and Future Directions

Several limitations should be considered when interpreting these findings. The main limitation was the small teacher sample size, with uneven distribution between groups, limited statistical power, and this may have increased the risk of Type II errors. Future studies should aim for larger, more balanced samples to enhance the robustness of findings. Second, although children were matched across intervention groups, significant baseline differences remained, particularly in child behavioral functioning. More stringent matching procedures or randomized designs in future research would strengthen causal inferences about JS intervention effects. Specifically, examining the relative contributions of the digital platform versus human consultation through a randomized controlled design would increase understanding of how to optimize the balance between technology and interpersonal support within IECMHC. Third, the intervention duration of 14 weeks may have been insufficient to detect certain outcomes, particularly those related to teacher well-being and self-efficacy, which may develop over longer timeframes. Longitudinal designs with extended follow-up periods would provide valuable insights into the sustainability of effects and patterns of app usage over time. Fourth, qualitative findings are subject to potential interviewer and coder bias. While double-coding and a structured analytic approach were used to enhance credibility, qualitative interpretations remain context-specific and may not be generalizable. Fifth, this study relied on teacher-reported measures, which introduces potential biases, including social desirability and recall bias. Although these reports offer valuable insight from key informants, future studies should incorporate multi-informant or observational data to strengthen measurement validity.

Lastly, while the technology acceptability measures provided important information about user perceptions, actual app usage data (e.g., frequency, duration, specific content accessed) was not systematically analyzed. Future studies should incorporate detailed app and AI analytics to understand how teachers engage with digital tools and which features most strongly predict implementation and outcomes. Further, refining the AI capabilities of the JS Go AI chatbot based on user interactions could enhance its relevance and effectiveness in addressing teachers’ specific needs as they evolve over time and potentially promote ongoing engagement.

Future research should explore the cost-effectiveness of hybrid consultation models compared to traditional approaches, particularly in terms of reach, impact, and sustainability in under-resourced settings. Economic analyses would provide valuable information for policymakers and funders considering investments in digital mental health infrastructure for early childhood mental health consultation systems.

## 5. Conclusions

Overall, this study provides initial evidence supporting the feasibility, utility, and potential efficacy of JS Go as an innovative approach to delivering IECMHC in early childhood settings. Despite methodological limitations, the findings suggest that integrating mobile technology with traditional consultation may offer a viable strategy for extending the reach of evidence-based mental health support to teachers and children. Children in both groups showed comparable and significant psychosocial gains, indicating that JS Go maintains the effectiveness of traditional IECMHC for child development. High teacher acceptability ratings further support the feasibility of digital platforms in early childhood settings, while qualitative findings highlight the value of pairing technology with interpersonal support. Notably, this study contributes to existing knowledge by demonstrating how hybrid models can deliver culturally responsive, scalable, and just-in-time consultation in an under-resourced, multilingual context. As early childhood programs continue to face significant challenges related to children’s behavioral health and teacher well-being, particularly in under-resourced communities, scalable solutions like JS Go represent a promising innovation in the mental health consultation landscape. This technology-enhanced approach not only extends the reach of evidence-based support but also empowers teachers with accessible, culturally and linguistically responsive resources to address challenging child behaviors and emotions in real time. Strengthening the mental health consultation infrastructure through hybrid models such as JS Go has the potential to enhance teacher capacity and improve children’s psychosocial outcomes, fostering more nurturing environments in which all young children can thrive. JS Go offers a viable path forward for expanding mental health consultation services in early learning environments. By offering accessible mobile support, this model has the potential to enhance teacher capacity, promote child well-being, and bridge longstanding equity gaps in access to early childhood mental health resources.

## Figures and Tables

**Table 1:** Qualitative Interview Guide for JS Go Directors and Teachers.

Q1: **[Effectiveness and Implementation]** Tell me about your experience with the JS Go program? How did the program help, if at all, you adopt JS Go practices? How are things different for you as a result of the program? How confident do you feel about using reflective practices with teachers in your program? What are some of the things that you have done, or will do, to use reflective practices with your teachers?
Q2: **[Effectiveness]** How do you think parents can use the JS Go information? Was information shared with parents? What, if anything, did the parents learn?
Q3: **[Effectiveness]** Tell me how confident you feel in your role as the JS Go director/teacher?
Q4: **[Effectiveness and Adoption]** Tell me about your experience working with your consultant around Using self-care strategies, such as creating a resiliency plan? Communicating with staff and families, such as having multiple modes of communication? Addressing racial equity topics such as not discriminating against someone by race and/or including all races and cultures in the program/classroom?
Q5: **[Implementation]** Tell me about your experience implementing Safety practices such as emergency procedures and policies? Behavioral supports to prevent challenging behavior and promote social emotional development, such as peer social skills?
Q6: **[Maintenance]** What do you need to maintain the JS Go program, specifically using the app on a regular basis, and use of the strategies in your center? What other resources for yourself and your staff? What other training for yourself and your staff?
Q7: **[Maintenance]** After reviewing the app’s gaming feature screenshots, What feedback do you have regarding the gaming screenshots? What additional features or changes would you suggest for the gaming feature? How helpful can this app be for helping teachers sustain their use of JS Go practices? How helpful can this app be for helping new teachers learn to use JS Go practices? What can facilitate the continuous usage of the app by the childcare center staff? Any other comments, questions, or concerns you would like to share about the app?
Q8: **[Adoption]** How interested would other staff in your center be in being a part of the JS Go program/working with the mental health consultant and using the app on a regular basis?
Q9: **[Implementation]** How well do you think your center is addressing the following: Safety Behavior Supports Self-Care Communication
Q10: **[Implementation]** What strategies do you think you can use to continue making improvements in Safety Behavior Supports Self-Care Communication

**Table 2:** Demographic Characteristics of Teachers by Intervention Group (JS Go vs. JS).

Variable	JS Go (*n* = 6)	JS (*n* = 22)	Test Statistic	*p*-Value
**Age (years)—M (SD)**	47.63 (8.52)	51.01 (11.95)	F(1,26) = 0.42	0.524
**Gender**			N/A	N/A
Female	6 (100.0%)	22 (100.0%)		
**Race**			Χ^2^(4) = 3.06	0.549
White	6 (100.0%)	14 (63.6%)		
Black	0 (0.0%)	4 (18.2%)		
Native American	0 (0.0%)	1 (4.5%)		
Multiracial	0 (0.0%)	1 (4.5%)		
Other	0 (0.0%)	2 (9.1%)		
**Ethnicity**			N/A	N/A
Hispanic	6 (100.0%)	22 (100.0%)		
**Primary Language**			N/A	N/A
Spanish	6 (100.0%)	22 (100.0%)		
**Education Level**			χ^2^(4) = 8.96	0.062
High School/GED	4 (66.7%)	2 (10.0%)		
Some College	0 (0.0%)	1 (5.0%)		
Associate Degree	0 (0.0%)	2 (10.0%)		
Bachelor’s Degree	2 (33.3%)	11 (55.0%)		
Graduate Degree	0 (0.0%)	4 (20.0%)		
**Completed Intervention**			χ^2^(2) = 1.37	0.505
Yes	5 (83.3%)	14 (63.6%)		
No	0 (0%)	4 (18.2%)		
Withdrew	1 (16.7%)	4 (18.2%)		
**Number of Consults for Completers M (SD)**	13.50 (1.23)	11.80 (2.15)	F(1,19) = 3.27	0.086
**Experience (years)—M (SD)**	6.56 (8.64)	9.76 (11.40)	F(1,24) = 0.40	0.533

*Note*. Missing data for education level (*n* = 2) in the JS group. Percentages are within the treatment group. χ^2^ = Pearson’s chi-square; N/A = not applicable as all participants were in the same category.

**Table 3:** Descriptive Statistics of Outcomes at T0 (Baseline) and T1 (Follow-up) by Intervention Group (JS Go vs. JS).

Outcome	JS Go			JS		
	Baseline Mean (SE)	Follow-Up Mean (SE)	Change	Baseline Mean (SE)	Follow-Up Mean (SE)	Change
HERS-C Safety	4.58 (0.20)	5.00 (0.18)	0.42	4.98 (0.27)	5.29 (0.57)	0.31
HERS-C Behavior	4.55 (0.19)	5.00 (0.29)	0.45	4.36 (0.54)	4.50 (0.43)	0.14
HERS-C Communication	4.00 (0.00)	4.30 (0.45)	0.3	4.36 (0.41)	4.43 (0.55)	0.07
HERS-C Resiliency	3.33 (0.52)	4.60 (0.55)	1.27	3.50 (1.16)	3.93 (0.27)	0.43
Brief Resiliency Coping	18.33 (1.86)	17.00 (1.79)	−1.33	18.05 (2.34)	17.86 (2.38)	−0.19
Teacher Opinion Survey	48.00 (5.51)	46.17 (2.93)	−1.83	44.76 (5.51)	45.79 (5.16)	1.03
CW Job Demands	2.78 (0.42)	2.74 (0.34)	−0.04	2.55 (0.99)	3.36 (0.58)	0.81
CW Job Resources	4.69 (0.42)	4.54 (0.34)	−0.15	4.48 (0.99)	4.45 (0.37)	−0.03
CW Job Control	3.63 (0.79)	3.06 (0.39)	−0.57	3.46 (0.89)	3.06 (0.67)	−0.4

*Note.* HERS-C abbreviated for Health Environment Rating Scale-Classroom. CW abbreviated for Childcare Worker.

**Table 4:** Results from ANCOVA Models Evaluating Treatment Group Effects at T1 (adjusting for T0).

Outcome	β (JS Go vs. JS)	SE	*p*-Value
HERS-C Safety	−0.39	0.15	0.0506
HERS-C Behavior	0.33	0.13	**0.0474**
HERS-C Communication	−0.49	0.21	0.07
HERS-C Resiliency	0.68	0.32	0.0876
Brief Resiliency Coping	−0.91	0.65	0.2226
Teacher Opinion Survey	−1.35	2.01	0.5331
CW Job Demands	−0.6	0.12	**0.0041**
CW Job Resources	−0.03	0.15	0.8272
CW Job Control	−0.07	0.24	0.7904

*Note.* HERS-C abbreviated for Health Environment Rating Scale-Classroom. CW abbreviated for Childcare Worker. Bold indicates statistically significant.

**Table 5:** Demographic Characteristics of Children by Intervention Group (JS Go vs. JS).

Characteristic	JS Go (*n* = 57)	JS (*n* = 57)	Total (*N* = 114)	*p*-Value
**Age in years**, *M* (*SD*)	3.70 (0.73)	3.83 (0.91)	3.76 (0.82)	0.436
**Gender**, *n* (%)				0.348
Female	28 (49.1%)	33 (57.9%)	61 (53.5%)	
Male	29 (50.9%)	24 (42.1%)	53 (46.5%)	
**Race**, *n* (%)				0.023 *
White	47 (88.7%)	41 (82.0%)	88 (85.4%)	
Black	0 (0.0%)	4 (8.0%)	4 (3.9%)	
Native American	2 (3.8%)	2 (4.0%)	4 (3.9%)	
Multiracial	4 (7.5%)	0 (0.0%)	4 (3.9%)	
Other	0 (0.0%)	3 (6.0%)	3 (2.9%)	
**Ethnicity**, *n* (%)				0.378
Hispanic	50 (94.3%)	47 (88.7%)	97 (91.5%)	
Non-Hispanic White	2 (3.8%)	2 (3.8%)	4 (3.8%)	
Haitian	0 (0.0%)	3 (5.7%)	3 (2.8%)	
Other	1 (1.9%)	1 (1.9%)	2 (1.9%)	
**English proficient**, *n* (%)				0.616
No	25 (47.2%)	22 (42.3%)	47 (44.8%)	
Yes	28 (52.8%)	30 (57.7%)	58 (55.2%)	

*Note.* Some categories have missing data, resulting in different sample sizes for analyses. Chi-square tests were used for categorical variables, and one-way ANOVA was used for age comparisons. * *p* < 0.05.

**Table 6:** Descriptive Statistics of Child Outcomes at T0 (Baseline) and T1 (Follow-up), by Intervention Group (JS Go vs. JS).

Outcome Mean (SE)	JS Go			JS		
	Baseline	Follow-Up	Change	Baseline	Follow-Up	Change
**DECA Attachment**	44.04 (1.39)	47.94 (1.39)	3.91	47.77 (1.10)	50.55 (0.96)	2.77
**DECA Initiative**	46.61 (1.22)	49.75 (1.51)	3.14	50.98 (1.33)	54.11 (1.18)	3.13
**DECA Self-Regulation**	50.53 (1.47)	54.02 (1.42)	3.49	52.68 (1.29)	54.20 (1.16)	1.52
**DECA Total**	46.81 (1.39)	50.54 (1.42)	3.73	50.81 (1.25)	53.92 (0.99)	3.12
**SDQ Externalizing**	6.79 (0.49)	6.35 (0.52)	−0.44	4.53 (0.60)	3.87 (0.51)	−0.65
**SDQ Internalizing**	5.30 (0.54)	1.87 (0.25)	−3.43	4.14 (0.58)	2.00 (0.34)	−2.14
**SDQ Total**	9.54 (0.76)	8.21 (0.64)	−1.33	6.93 (0.89)	5.87 (0.78)	−1.06

*Note.* DECA is abbreviated for Deveraux Early Childhood Assessment. SDQ is abbreviated for Strengths and Difficulties Questionnaire.

**Table 7:** Generalized Estimating Equation (GEE) Results for Child Outcomes.

Outcome	Time Effect (Follow-Up vs. Baseline) β (SE)	*p*-Value (Time)	Treatment Group (JS Go vs. JS) β (SE)	*p*-Value (Treatment)	Interaction (Time × Treatment) β (SE)	*p*-Value (Interaction)
**DECA Attachment**	2.89 (1.27)	**0.0226**	−3.74 (1.76)	**0.0335**	0.79 (1.78)	0.658
**DECA Initiative**	3.05 (1.20)	**0.0108**	−4.37 (1.79)	**0.0145**	−0.26 (1.85)	0.8896
**DECA Self-Regulation**	1.78 (1.29)	0.1666	−2.16 (1.94)	0.2651	1.20 (2.01)	0.5499
**DECA Total**	3.22 (1.22)	**0.0084**	−4.00 (1.85)	**0.0307**	0.14 (1.88)	0.9421
**SDQ Externalizing**	−0.74 (0.50)	0.141	2.26 (0.77)	**0.0033**	0.61 (0.67)	0.3642
**SDQ Internalizing**	−2.18 (0.51)	**<0.0001**	1.16 (0.78)	0.1385	−1.09 (0.71)	0.1253
**SDQ Total**	−1.16 (0.74)	0.119	2.61 (1.16)	**0.0243**	0.33 (0.98)	0.7369

*Note.* DECA is abbreviated for Deveraux Early Childhood Assessment. SDQ is abbreviated for Strengths and Difficulties Questionnaire.

**Table 8:** Descriptive Statistics for JS Go Technology Acceptability and App Quality Measures.

Variable	N	Minimum	Maximum	Mean	Standard Deviation
**Technology Fast Form**					
Usefulness	6	1	4	3.56	1.01
Ease of Use	6	2	4	3.67	0.82
Predicted Future Usage	6	−4	4	1.38	3.63
**mHealth App Usability Questionnaire**					
Ease of Use	6	5	7	6.43	0.59
User Interface	6	6	7	6.52	0.46
Usefulness	6	6	7	6.47	0.45
**Mobile App Rating Scale**					
Engagement	6	4	5	4.73	0.24
Functionality	6	4	5	4.79	0.51
Aesthetics	6	4	5	4.72	0.53
Information	6	4	5	4.67	0.42
Subjective App Quality	6	4	5	4.73	0.38

## Data Availability

Requests for data can be sent to the corresponding author.

## Abbreviations

The following abbreviations are used in this manuscript:

AI	Artificial intelligence
ANCOVA	Analysis of covariance
CWJSI	Childcare Worker Job Stress Inventory
DECA	Devereux Early Childhood Assessment
ECE	Early care and education
FF-TAM	Technology Acceptance Model Instrument-Fast Form
GEE	Generalized estimating equation
HERS-C	Health Environment Rating Scale-Classroom
IECMHC	Infant and early childhood mental health consultation
JS	Jump Start
JS Go	Jump Start on the Go
MARS	Mobile App Rating Scale
MAUQ	mHealth App Usability Questionnaire
MHC	Mental health consultant
REDCap	Research Electronic Data Capture
RQA	Rapid qualitative analysis
SDQ	Strengths and Difficulties Questionnaire
TPF	Total Protective Factors
